# Analysis of mutation, selection, and epistasis: an informed approach to cancer clinical trials

**DOI:** 10.18632/oncotarget.25155

**Published:** 2018-04-27

**Authors:** Jon F. Wilkins, Vincent L. Cannataro, Brian Shuch, Jeffrey P. Townsend

**Affiliations:** ^1^ Ronin Institute, Montclair, NJ, USA; ^2^ Department of Biostatistics, Yale School of Public Health, Yale University, New Haven, CT, USA; ^3^ Department of Urology, Yale School of Medicine, New Haven, CT, USA; ^4^ Department of Radiology, Yale School of Medicine, New Haven, CT, USA; ^5^ Department of Ecology and Evolutionary Biology, Yale University, New Haven, CT, USA; ^6^ Program in Computational Biology and Bioinformatics, Yale University, New Haven, CT, USA

**Keywords:** cancer, natural selection, evolution, mutation, epistasis

## Abstract

Currently, drug development efforts and clinical trials to test them are often prioritized by targeting genes with high frequencies of somatic variants among tumors. However, differences in oncogenic mutation rate—not necessarily the effect the variant has on tumor growth—contribute enormously to somatic variant frequency. We argue that decoupling the contributions of mutation and cancer lineage selection to the frequency of somatic variants among tumors is critical to understanding—and predicting—the therapeutic potential of different interventions. To provide an indicator of that strength of selection and therapeutic potential, the frequency at which we observe a given variant across patients must be modulated by our expectation given the mutation rate and target size to provide an indicator of that strength of selection and therapeutic potential. Additionally, antagonistic and synergistic epistasis among mutations also impacts the potential therapeutic benefit of targeted drug development. Quantitative approaches should be fostered that use the known genetic architectures of cancer types, decouple mutation rate, and provide rigorous guidance regarding investment in targeted drug development. By integrating evolutionary principles and detailed mechanistic knowledge into those approaches, we can maximize our ability to identify those targeted therapies most likely to yield substantial clinical benefit.

The sequencing of the human genome at the beginning of this century ushered in a revolution in genomic medicine [1, 2], and many promising therapeutic approaches are in development that make primary use of a patient's genotype data, such as genetic markers in tumor cells. The synergistic combination of personalized medicine with molecular therapeutics that target specific cancer drivers has yielded some remarkable successes, but also a string of disappointments [3–5]. More than thirty targeted cancer therapeutics have been approved for clinical use, but the efficacy of these new drugs has proven variable, with limited durable responses seeming to depend on tumor type and secondary biomarkers [6–12]. Therapeutics that specifically target a single gene product—and especially those that target the mutant, oncogenic form—have the potential to produce fewer unwanted effects on non-tumor cells relative to conventional chemotherapeutics that target all dividing cells. For targeted therapeutics to achieve optimal effectiveness, though, requires accurate application of personalized medicine. If a drug targets a specific genetic variant, it will likely only benefit a patient whose tumor is carrying that variant, and in whom that variant is driving tumorigenesis.

Although this “matching” of targeted therapeutic to a precision-medicine profiled patient is straightforward—in principle—the list of potential molecular targets (and drugs targeting them) continues to expand [13, 14], as does our understanding of the frequency at which specific genetic variants are found in various cancers [15, 16] and our understanding of the genetic heterogeneity of tumors [17–19]. This expansion of targets across cancer types naturally generates a huge number of plausible hypotheses: specific patient populations who might benefit from specific targeted therapies, or combinations of therapies [20, 21]. As it is not possible to test every mutation type in every cancer, a tissue-agnostic approach bringing an agent forward to the clinic may be necessary once there is strong preclinical rationale that inhibiting a specific pathway has efficacy in a different cancer model. Testing the hypothesis of efficacy based on a different preclinical model with strong laboratory and genomic evidence still requires subsequent clinical trials [22].

As the numbers of candidate therapies and putative targets increase, the development of theoretical frameworks providing rational, quantitative methods for identifying drug/target combination becomes increasingly important. The number of ideas that can be defended on the grounds of plausibility is large, but the fraction of these ideas that result in superior therapies is likely to be small. Not only considerations of cost- and time-effectiveness, but also moral considerations regarding patient welfare, demand that we develop approaches that have predictive value regarding the potential success or failure of targeted drug development [23], derived from what we know about the genetic architecture of cancer.

While precision medicine tumor boards and programs like NCI-MATCH [24] explicitly aim to pair patients with treatment options based on the tumor genomic profile, we feel that these and future efforts can be enhanced by informing decisions with all available sources of data and insight on cancer progression and treatment. One such additional source of information potentially derives from taking an evolutionary perspective on tumor progression [25, 26], employing tools and insights from population genetics. In this paper, we discuss the value and importance of considering the strength of selection favoring the persistence and spread of specific high-fitness genetic variants within the context of the heterogeneous tumor environment. “Selection” or “fitness” of somatic genetic variants in this context refers to the differential survival and reproduction of cell lineages within the patient, and not the fitness of the patient. Estimates of the strength of this selection suggest an upper bound for the potential efficacy of therapies targeting oncogenic mutations, before pre-clinical data can be obtained that provides pharmacokinetic guidance or biologically effective doses (e.g.[27, 28]). We also discuss how estimates of selection will be affected by variation in the oncogenic mutation rate and by epistasis among oncogenic mutations.

For newly developed targeted therapies, it is tempting to focus on oncogenic targets found in a high fraction of patients (although some drugs work well for targets found in a low fraction). This approach is sensible not only because there is a larger patient population available and because the required accrual for a clinical trial is easier, but also for effectiveness and impact. First, a variant found at high frequency in a particular type of tumor is likely to play a causal role in tumor growth. Second, should the clinical trial prove effective, a therapy targeting a more common variant will have a greater downstream impact on public health. For example, mutations in the *BRAF* gene are found in over 50% of melanomas [29], which made this cancer an attractive candidate for clinical trials on therapies that target *BRAF*-mutant tumors via the oncogenic variant gene product. One decision rule for the execution of novel clinical trials is to test therapies that have proven effective against one cancer by applying them to other tumor types [30]. As mentioned above, there may not be a sufficient preclinical model to test the efficacy in the laboratory for a particular cancer in which extensive testing suggests benefit. In this situation, there may be merit to a tissue-agnostic approach, as it focuses on drugs with *in vivo* anti-tumor activity and known side effects, in contrast with early stage drugs with unknown safety profiles. But if an anti-*BRAF* therapy proves effective against melanoma, should we expect that effectiveness to carry over to patients with *BRAF* mutations in other cancers in which *BRAF* mutations are found at much lower frequencies?

The “causal-role” logic of the argument to perform clinical trials that focus on therapeutics targeting high-frequency somatic variants argues against trials of their efficacy in cancers in which the variants are rarely found. But there is more guidance that can be derived from the somatic genetic architecture of cancer types than this simple logic would dictate. The frequency at which genetic variants are found in particular cancers is determined by two factors: 1) the rate at which oncogenic mutations arise, and 2) the effect of natural selection on those mutations within the population of cancerous (or pre-cancerous) cells. Both mutation rate, which varies with gene location [31] and gene expression levels [32], and mutation target size, vary tremendously across the genome [16]. Strength of selection is also enormously variable across the genome and ontogeny [33], highly dependent on the tissue type and cellular environment (including the presence of anti-cancer therapy), and complicated by epistatic interaction among oncogenic variants [34]. Decoupling the contributions of mutation and selection to the frequency of somatic variants is critical to understanding—and predicting—the therapeutic potential of different interventions within individual cancer types. The potential benefit of a therapeutic will relate to the strength of selection favoring the oncogenic variants, and will generally be unrelated to the rate at which the oncogenic mutations occur. However, the observable data—the frequency of a variant in the context of a given cancer—is affected by both the strength of selection and rate of mutation. Thus, estimations of selection, and the associated predictions regarding therapeutic benefit, require an appropriate accounting for any variation in the mutation rate.

## VARIATION IN THE ONCOGENIC MUTATION RATE

The per-gene mutation rate varies across the genome by more than an order of magnitude. This extensive variation is attested to and accounted for by established tools like MutSigCV [16] that aim, by correcting for the mutation rate, to help identify the genes burdened with mutations in a higher-than-expected fraction in a particular cancer. The use of a well-estimated mutation rate to identify genes contributing to cancer represents an important first-order correction necessary for disentangling the effects of mutation and selection. In addition to the variation in mutation rate across the genome, there is substantial variation in the rates of specific types of mutations by nucleotide and cell type. For instance, G→A and C→T transitions are more common than A→T and T→G transversions due to biochemical properties of the nucleotides, and lung cancers have a characteristically high G→T transversion incidence attributed to tobacco-specific mutagens [35].

Equally important for decoupling the effects of mutation and selection is understanding the variation in the oncogenic mutation target size. For genes where loss of function is oncogenic, there will be a variety of missense, nonsense, deletion, and insertion mutations that will disrupt gene function and drive tumorigenesis. However when the druggable target is the result of an activating missense mutation, activation likely depends on a small number of very specific mutations. For example, of the approximately 50% of melanoma patients whose tumors carry mutations in *BRAF*, nearly 90% have a substitution at amino acid position 600, and 90% of these substitutions are V600E, which occurs only from a nucleotide position 1799 T → A substitution [36]. Transition mutations occur at roughly twice the frequency of transversions [37]. At position 1799 T, approximately 50% of new mutations would be T → C transitions while T → G and T → A transversions would account for 25% each. Thus, the most common substitution, observed in over 40% of melanomas, results from the least probable point mutation at that single nucleotide position. This combination of high frequency and mutational rarity implies that the V600E substitution must confer a high selective advantage in melanoma tumor cell populations.

Other oncogenic mutations have a broader distribution of substitutions at a single site. For instance, approximately 20% of melanoma patients have tumors carrying mutations in *NRAS*, and of these approximately 80% have amino acid substitutions at position 61, including Q61R (38%), Q61K (24%), and Q61L (15%) [38]. Like *BRAF* V600E, the Q61K and Q61L substitutions result from transversion mutations (181 C → A and 182 A → T, respectively). The Q61R substitution results from a transition mutation (182 A → G). Thus the *BRAF* V600E substitution is observed at twice the frequency of *NRAS* Q61 substitutions in melanoma—despite the fact that oncogenic mutations at *NRAS* Q61 likely arise at a higher frequency, both because there are more oncogenic mutations available (Q61K, Q61L, and Q61R, *versus* V600E), and because one of those mutations relies on the more frequent transition process. These ratios imply that the impact of selection on the frequency of observing the *BRAF* V600E substitution is many times greater than the impact of selection on the frequency of the common oncogenic *NRAS* Q61 substitutions. The relative maximum therapeutic benefits of targeting *BRAF* and *NRAS* should scale similarly.

This example comparing *BRAF* and *NRAS* in melanoma illustrates a general point that, in addition to the variation among genes in the per-nucleotide mutation rate, there are at least two other sources of variation that affect what we might call the “oncogenic mutation rate,” meaning the rate of occurrence of mutations that actually contribute to cancer growth. The first source of variation is the number of distinct substitutions that result in an oncogenic phenotype. The second is the difference in rates of different types of mutational processes (e.g., transitions *versus* transversions). The frequency at which a gene is observed as a high-frequency variant in tumor tissues of a given cancer depends both on the rate at which oncogenic mutations occur and on the selective advantage that they confer. For a variant occurring at a given frequency, the rarer the mutation, the greater the selective advantage the alteration must have conferred. The potential for variation in the oncogenic mutation rate becomes even more pronounced when we consider mutation processes other than nucleotide substitutions, such as deletions that remove entire exons, or translocations that give rise to specific fusion proteins. The rates at which these other types of mutations occur are generally less well known than nucleotide substitution rates, but understanding them is critical for our interpretation of the frequencies at which the resulting variants are found in different cancers.

## SELECTION, THERAPEUTIC POTENTIAL, AND EPISTASIS

If a particular mutation seldom occurs, yet is found at high frequency among cancers, this contrast implies that the mutation confers a strong positive selective advantage to the cells possessing it. An intervention that specifically targets that mutation, and thereby removes that selective advantage, would have a substantial therapeutic impact. The mutation rate for the *BRAF* V600E mutation described above is very low, but the mutation is nonetheless present in tumors at an incommensurate frequency in primary skin cutaneous melanoma and colon adenocarcinoma, where it is a known driver, and in low-grade glioma and lung adenocarcinoma where it has been calculated to be intensely selected [39]. It is not surprising then, that therapies that specifically target that variant are particularly effective for patients with melanoma [40], at least in the short term. At the other end of the spectrum are examples like *TTN*, which, with its ~33,000 amino acids, is the largest known human protein. *TTN* has shown up in many tumor sequencing projects as a putative cancer driver but, as a structural component of muscle sarcomeres, is widely discredited as such. Rather, its extreme size means that it presents an extraordinarily large mutational target; tumors will often contain one or more *TTN* mutations, even if little or no selection is involved [16]. The extent to which natural selection favors a given oncogenic variant is directly connected to that variant's contribution to cancer progression. Within a population of cancer (or pre-cancerous) cells, those genotypes that lead to the cell dividing more frequently—and those that lead to the cell undergoing cell death or terminal differentiation less frequently—increase in frequency in the same kind of evolutionary process that leads to the spread of fitness-enhancing variants in cellular populations. The fitness associated with a new mutation is directly related to its enhancement of the cancer phenotype within its current environment. And the frequency at which we observe a given variant across patients—modulated by our expectation given the mutation rate and target size—is an indicator of that strength of selection. That is, an oncogenic driver mutation is simply a “beneficial” mutation that is under positive selection within the population of tumor cells.

**Figure 1:**
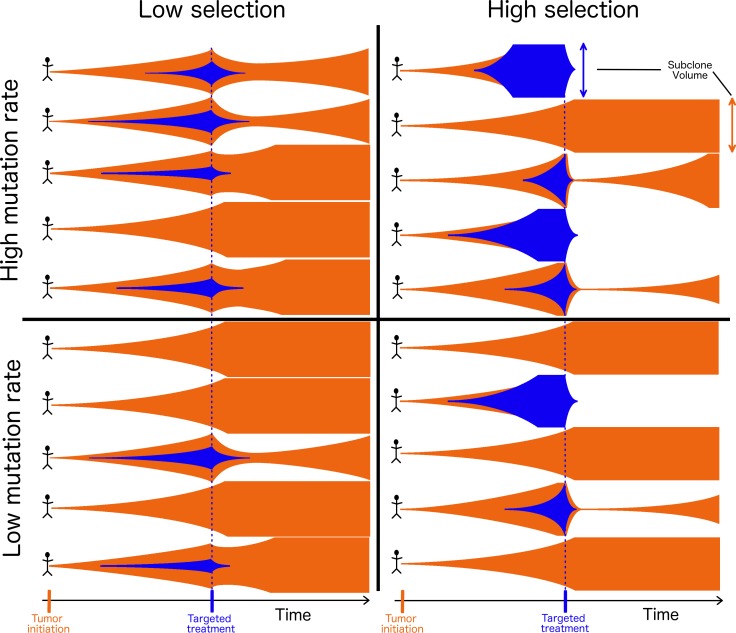
Tumor evolution and treatment under high and low mutation and high and low selection scenarios Each tumor is initiated by an original cell population (orange), and populations expand in size (vertical axis) over time (horizontal axis). As the tumor grows, a new subclone (blue) can be founded via a mutation that confers a more extensive cancer phenotype, and can grow alongside or supplant the original population. Targeted treatment (blue line) specifically eliminates the new population that it targets (blue), leading to a varying consequence on the tumor that depends on the extent of intra-tumor heterogeneity, which is determined by its selective effect. Tumors with a high proportion of their cells belonging to the population affected by treatment are most likely to experience the greatest reduction of tumor burden, the longest remission before recurrence, and the greatest opportunity for cure.

Tumors exhibit some degree of intra-tumor heterogeneity. It is this heterogeneity that provides the context within which selection can act; the greater fitness conferred by particular mutation, the more common it will become within that heterogeneous population. Put another way, if we observe that a particular variant is very common among patients with a tumor type—and/or very common within heterogeneous tumor-cell populations—this high frequency suggests that the variant has a large effect on the ability to grow, divide, survive and indeed, to contribute to any of the hallmarks of cancer [41] other than increasing mutation rate. Consequently, if we have a therapeutic agent that inactivates that variant, we expect it to have a correspondingly large and negative impact on cancer cells within the tumor. If, however, that same variant is rare in a second tumor type, then that is an indication that the mutation provides less of a selective advantage in that cell type. Consequently, counteracting that mutation with a targeted drug would likely have a less severe impact on the ability of cancer cells to thrive, and a lower salutary impact on the patient (Figure 1). Clearly, for heterogeneous tumors, the efficacy of any targeted therapy will be limited to the subset of cells expressing the appropriate target. Thus, targeting oncogenic driver mutations that are more strongly favored by selection will also tend to lead to the greatest reductions in tumor volume.

The strength of selection favoring a particular genetic variant will predict not only the potential efficacy of targeted therapeutics, but also the strength of selection favoring the evolution of resistance following the application of those therapeutics [42]. The fitness of the mutation within the tumor environment is dependent on the context within which mutations and therapies interact. The introduction of any therapy alters that landscape, creating new selective pressures favoring mutations that interfere with the therapy or bypass its locus of action [43]. Moreover, other factors can modulate the selective advantage conferred by a mutation. For instance, the *BCR-ABL1* fusion is present in 90–95% of patients with chronic myeloid leukemia [44]. This fusion arises from a t(9;22) (q34.1;q11.21) translocation that results in the Philadelphia chromosome [45]. While the exact rate at which this translocation spontaneously occurs is unknown, it appears that it occurs frequently in white blood cells of healthy individuals [46]. The translocation is considered a major driver of chronic myeloid leukemia, apparently because as cellular fitness declines with age or potentially other influences, the translocation becomes increasingly strongly favored by natural selection [47, 48]. It would be no coincidence, then, that therapeutics specifically targeted against the BCR-ABL fusion protein (e.g., imatinib) have provided an outstanding example of successful targeted chemotherapy, potentially providing patients with long-term health outcomes similar to those of the general population [49, 50]. The substantial rate of evolution of resistance and recurrence is consistent with imatinib imposing a strong selective pressure.

The strength of natural selection favoring each genetic variant in each cancer type can be inferred using theoretical approaches and tools derived from population genetics [39, 42]. Then it can be used as a predictor for the efficacy of therapeutics targeting specific oncogenic variants in different cancers or in individual molecular subsets of a cancer type defined by epigenetic states or cellular context [51]. However, estimates of selection intensity based solely on somatic variant frequency and mutation rate will have to be interpreted with caution, due to the complicating effects of epistasis—the phenomenon in which the strength of selection acting on a mutation depends on the presence or absence of other mutations elsewhere in the genome. Epistasis is known to be present among causal somatic variants in cancer, usually identified in cancer genomics by mutual exclusivity or co-occurrence of mutations [52–55]. However, statistical identification of epistatic interactions typically requires strong epistasis and large sample sizes. Fortunately, mechanistic knowledge of the biochemical interactions in the cell and the signaling pathways where dysregulation often leads to cancer provides a way to focus a search for epistasis in principled ways [55, 56]; in turn, knowledge of epistatic interactions can serve as a further guide to the potential efficacy of a targeted therapeutic agent. This knowledge can facilitate more accurate characterization of selection strengths acting on individual genes and a better understanding of how those selection strengths vary based on other genetic markers.

To understand the effect of epistatic interactions on the potential efficacy of a targeted therapeutic, it will help to categorize interactions as either “antagonistic” or “synergistic”. When the selective advantage of two mutations is less than expected from the advantages conferred by the single mutations, the interaction is antagonistic. Antagonistic epistasis affecting selection on oncogenic mutations manifests in patterns of mutual exclusivity. Two different mutations may be favored by selection separately. However, with antagonistic epistasis, the presence of one mutation reduces or eliminates the selection favoring the other one. As a result, while we would find each mutation at high frequency in cancer cells, rarely would we find both mutations in the same cell. Biochemically, antagonistic epistasis can arise when one gene product is downstream from another in a signaling pathway—though other antagonistic interactions are known [57].

**Figure 2:**
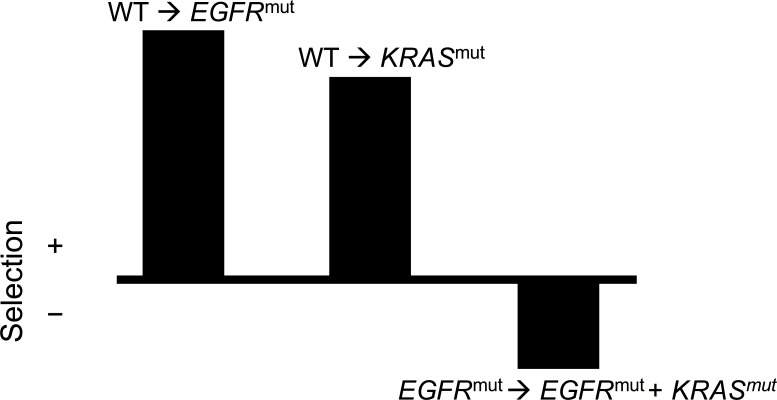
Illustration of negative epistasis between oncogenic EGFR and KRAS mutations in lung tissue Individually, EGFR and KRAS mutations both confer a survival and/or reproductive benefit to lung cells, and are thus positively selected. However, if a cell contains both oncogenic mutations, there is a decline in survival and/or reproduction. Therefore, the double mutant is subject to negative selection—in this case, an epistatic effect of the possession of one mutation upon the selection imposed on the second mutation.

One example is the interaction between activating mutations of *EGFR* and downstream *RAS* genes. Oncogenic mutations in these genes result in constitutive activation of a mitogenic signaling pathway, but only one of the two mutations is required to achieve an oncogenic effect. If a mutation has activated *EGFR*, activating *RAS* mutations have little additional growth effect, and do not spread through the population of cancer cells. Likewise, if a downstream *RAS* becomes constitutively active and no longer dependent on upstream activation, mutations in *EGFR* will exert no downstream effect, and no selective advantage. For example, in lung adenocarcinoma, ~30% of tumors carry a mutation in the *KRAS* gene and ~15% carry a mutation in *EGFR*, but these mutations are mutually exclusive [58, 59]. If antagonistic epistasis is sufficiently strong, the presence of both mutations together can actually be deleterious (disfavored by selection), even though each is beneficial (favored by selection) individually (Figure 2). In fact, there is evidence from transgenic mouse models and cancer cell lines that simultaneous activating mutations in *KRAS* and *EGFR* lead to cell death in lung adenocarcinoma [60]. Despite differences in their roles in signalling and in therapeutic implications, a similar mutually exclusive pattern of mutation extends to KRAS and downstream BRAF [61], and extends further down mitogen-activated protein kinase pathways.

Existing methods can estimate the intensity of selection acting on specific genes and variants within a particular cancer type [39, 42]. The most straightforward application of those methods—and possibly the only statistically supportable one—estimates selection under the assumption that there is no epistasis. To the extent that antagonistic epistasis is present, assuming it is not would lead to underestimation of the level of selection on the antagonistic mutations and thus underestimation of the potential clinical benefit of targeted therapy. Assuming no epistasis effectively yields averages over the different genetic contexts in which the mutation may arise: weak or even negative selection in the presence of the epistatic partner and strong positive selection in the absence of that partner. However, under antagonistic epistasis, most or all tumor tissues with one somatic variant of an epistatic pair will lack the partner variant, leading to a situation where no matter what the selection coefficient on acquisition of the partners are, neither can reach the highest frequencies across tumors that mutations without antagonistic epistasis can. The somatic variant frequency across tumors stalls at a virtual “ceiling” created by the incidence of its epistatic somatic variant; the strength of selection will be underestimated, even after fully accounting for the oncogenic mutation rate. The clinical benefit of effective targeted therapy for these patients will be enhanced relative to the benefit predicted from the average selective strength inferred under an assumption of no epistasis.

Patterns of antagonistic epistasis or mutual exclusivity can furthermore provide insight into possible modes of drug resistance due to selective pressure by a targeted agent [62]. These mutational patterns indicate alternative molecular mechanisms for achieving the same oncogenic cellular phenotype. For example, *RAS* mutations (most commonly at the G12 and G13 sites) represent a common form of evolved resistance to therapies targeting *EGFR* in metastatic colorectal cancer [63]. The strong positive selection on *EGFR*-activating mutations predicts a substantial clinical response to treatments targeting the activated protein. However, due to epistasis, this treatment has a side-effect of altering the strength of selection on other genes—e.g., making activating *RAS* mutations that would have been lethal in the presence of uninhibited EGFR become strongly beneficial for the cancer lineage(s) in the presence of targeted EGFR treatment. Thus, understanding the patterns of epistasis in these cells can not only provide a better ability to identify potential drug targets, but also to predict the likely modes of resistance to future therapies.

In contrast to antagonistic epistasis, the selective advantage of two mutations can also be greater than expected from the advantages conferred by the single mutations, in which case the interaction is synergistic. Synergistic epistasis in cancer manifests in patterns of co-occurrence. In the case of two synergistic mutations, each of them on their own is favored only modestly by selection (and they are only modestly oncogenic). But when both mutations are present together, selection on the pair is strong. That is, selection strongly favors one mutation only in the presence of the other. Thus, finding either mutation alone will be rare, but the pair of mutations will be highly enriched. The first case of synergistic epistasis described for cancer was between RAS and MYC, when researchers demonstrated that full tumorigenic conversion of fibroblasts required introduction of both oncogenes [64, 65]. Other genes have been found to also interact synergistically with RAS, as oncogenes promoting hyper-proliferation such as RAS can also promote cell-induced senescence [66], and negation of this senescence by other mutations in genes, such as p53, exacerbates cellular proliferation [67]. A similar synergistic mechanism involving p53 was also discovered for BRCA2 [68] and BRCA1 [69], FGFR2 [70] and for amplification of other genes [71] including ERBB2. This well-described mechanistic epistasis can give rise to a more abstract statistical epistasis: mutations in *TP53* are found nearly twice as often in ovarian cancers with *BRCA1* or *BRCA2* mutations compared with tumors with wild-type *BRCA1* and *BRCA2* [72, 73]. This pattern of co-occurrence is a consequence (and an indicator) of the fact that the selective advantage of an loss-of-function *TP53* mutation depends on the genotype at these other loci. We would expect that loss-of-function mutations in *TP53* and other apoptosis genes should be positively epistatic (synergistic) with activating mutations in oncogenes.

As was the case with antagonistic epistasis, the presence of synergistically epistatic interactions means that targeted therapies could provide greater clinical benefit to the appropriately targeted population than would be predicted on the basis of estimates of selection that assume no epistasis. An estimate of selection on *TP53* would average over cases with and without *BRCA* mutations. Therapies targeting *TP53*-mutant cells would be most effective in cases where those cells also carried mutations in *BRCA1* and/or *BRCA2*. Tumors exhibiting a *TP53* mutation would be enriched for exactly those mutations. The patient population toward which such a targeted treatment should be directed would constitute those who exhibit the common pattern of co-occurrence.

Synergistic epistasis is at the core of the multiple-hits model of cancer progression. Implicit in the model that you need a certain number of mutations before you get cancer progression is the idea that selection on individual variants is weak, and only becomes strong when multiple oncogenic variants are present simultaneously. The model also implies that targeting any one of causal variants should be sufficient to yield a beneficial response—although targeting multiple co-occurring variants might reduce the opportunity for the evolution of resistance to therapy. Similarly, synergistic epistasis may also play a role in “cancer fields”, where collections of cells carry potentially oncogenic variants, but do not develop into full-blown cancer until one or more additional mutations arise. In this case, weak selection on the pre-cancerous field cells may contribute modestly to their expansion, but selection does not become strong until an epistatic partner arises through mutation.

Antagonistic and synergistic epistasis not only have distinct implications for the efficacy of therapy, but also often reflect different sorts of biochemical relationships in the cell. Antagonistic epistasis can arise between genes in linear or linear-like pathways with upstream-downstream relationships, whereas synergistic epistasis can arise between genes with partially overlapping or redundant functionality. Nevertheless, the two forms of epistasis have similar implications for how we should interpret the relationship between estimates of the strength of selection favoring an oncogenic variant and the potential clinical benefit of therapies targeting that variant. Due to limitations imposed by small sample sizes, it is often viewed as unavoidable in analyses of genome-scale data to assume that there is no epistasis. However, for variants that are subject to strong epistasis (antagonistic or synergistic), the selective advantage in the clinically relevant genetic context will be greater than a no-epistasis estimate would indicate. Thus, the potential clinical benefit of therapeutic targeting of genes that have epistatic partners is higher than would be predicted by an analysis that assumes the effects of therapeutic targeting of each gene are independent.

## CONCLUSIONS

The development of increasing numbers of cancer therapeutics designed for specific molecular targets can provide significant efficacy in appropriately selected populations, as well as a reduction in side effects. However, this burgeoning multiplicity of treatment options simultaneously leads to a large number of potential combinations of therapies that each can be tested on a large number of classifications of tumor types often where hypothesis testing may not be feasible in the laboratory setting. When sufficient preclinical evidence exists for efficacy with a useful agent, each novel therapy or combination of therapies will need to be validated in clinical trials before general adoption, putting high strain on the resources required for drug development and for conducting clinical trials. Ethical considerations for the well-being of patients and financial considerations arising from the high up-front costs of a well-powered clinical trial demand that we develop systematic approaches that have predictive power to identify those therapies and therapeutic combinations that are most likely to be successful.

When treatments site-specifically target and inhibit a gain of function caused by an oncogenic mutation the potential of a targeted therapy is linked to the capacity of a targeted variant to drive tumor growth. That capacity is related most directly not to the frequency of that variant among tumors, but rather to the selective advantage associated with the oncogenic variant. Provided that an effective and tolerable pharmacological agent can be developed, those variants that provide the greatest selective advantage provide the greatest potential as promising therapeutic targets. Disruption of these targets will have the greatest impact on the growth and survival of tumor cells. To accurately estimate the selective advantage of oncogenic mutations requires accounting not only for the variant frequency across tumors, but also for variation in the underlying oncogenic mutation rates. Mutation rates vary not only across the genome and but also among different types of mutation. Moreover, genes vary in the number and types of mutations that will give rise to oncogenic variants. Accurately accounting for these sources of variation will substantially enhance our ability to identify and target causal oncogenic variants.

Attempts to estimate selection are also complicated by antagonistic and synergistic epistasis, which are likely widespread among oncogenes and tumor suppressors. Due to limited sample sizes, epistasis is often challenging to identify conclusively on purely statistical grounds. However, knowledge of the biochemical relationships among gene products of interest can help to identify likely epistatic interactions. Where those biochemical relationships suggest epistasis, the selective advantage of oncogenic variants and the therapeutic opportunity is likely to be much greater than would be expected by genomic analyses of underlying mutation rate and variant frequency alone. Thus, all else being equal, a selectively favored molecular target—especially one suspected of having strong epistatic interactions—represents a promising candidate. Quantitative approaches need further development that use the known genetic architectures of cancer types to provide rigorous guidance regarding investment in prospective clinical trials. By integrating evolutionary principles and detailed mechanistic knowledge into those approaches, we will be able to maximize our ability to identify those combinations of targeted therapies and cancer types most likely to yield substantial clinical benefit.
